# The interplay between cultural models and metaphor understanding: a cross-cultural cognitive perspective

**DOI:** 10.3389/fpsyg.2025.1539784

**Published:** 2025-06-25

**Authors:** Jun Zou, Carol Fuller, Linyao Wang

**Affiliations:** ^1^School of Foreign Languages, Shaoxing University, Shaoxing, China; ^2^Institute of Education, University of Reading, Berkshire, United Kingdom

**Keywords:** metaphor, cultural model, cross-cultural, key elements, grounded theory

## Abstract

In cross-cultural communication, accurate metaphor comprehension enhances mutual understanding and facilitates effective cooperation among individuals from diverse cultural backgrounds. This paper adopts a cross-cultural cognitive perspective and employs grounded theory as the methodological framework to analyze how cultural models influence metaphor understanding. Through a coding analysis of 148 domestic and international texts, the study constructs a four-element model comprising thinking patterns, cognitive frameworks, language communication, and social consensus. Thinking patterns shape cognitive frameworks, which are articulated and transmitted through language communication, ultimately contributing to the formation of social consensus within cultural groups. These four interrelated elements work together to support deeper and more accurate metaphor comprehension in intercultural contexts. By integrating theory with empirical analysis, this study offers a novel conceptual framework for future research on metaphor in cross-cultural communication.

## 1 Introduction

In an era of accelerating globalization, cultural exchange has become an essential driver of mutual understanding and cooperation among nations. Particularly in Asia, initiatives such as China’s active promotion of international cultural dissemination underscore the importance of mutual learning among civilizations (State Council Gazette). These efforts not only enhance global cultural influence but also shape new metaphorical understanding patterns that extend beyond linguistic expression to become key cognitive and communicative tools (Li, 2021b; Li, 2021a).

Metaphor, as a fundamental mechanism of human cognition and communication, carries deeply embedded cultural connotations. It enables individuals to frame abstract concepts through culturally shaped imagery and reasoning (Lakoff and Johnson, 1980). In cross-cultural contexts, metaphor functions not only to convey implicit meanings but also to mediate cultural values and worldviews (Kövecses, 2005; Musolff, 2014). However, when metaphorical expressions are interpreted solely within the confines of a single cultural framework, they risk being misunderstood, leading to communicative friction or cultural estrangement. This highlights the urgent need for more nuanced metaphor studies that consider the diversity of cultural models across languages and societies.

While previous research has explored the cognitive and linguistic aspects of metaphor, limited attention has been given to the systematic interplay between cultural models and metaphor understanding in a cross-cultural context. For example, studies by Yu (2007) and Deignan (2003) point out cultural variations in metaphor use but often lack comprehensive theoretical frameworks to explain how these variations influence comprehension across cultures. Moreover, there remains a gap in empirical studies that integrate cultural cognition with metaphor interpretation through grounded research methods.

To address this gap, the present study adopts a cross-cultural cognitive perspective and employs grounded theory to analyze 148 domestic and international texts discussing cultural models and metaphor use. It aims to identify and conceptualize the key elements involved in metaphor comprehension across cultures—namely, thinking patterns, cognitive frameworks, language communication, and social consensus. These elements are examined as dynamic components of cultural models that influence how metaphors are interpreted and internalized in different cultural settings.

By constructing a theoretical model grounded in empirical data, this study not only advances our understanding of metaphor in cross-cultural communication but also contributes practical insights for addressing cultural misunderstandings. It seeks to enrich the discourse on metaphor and culture by offering a systematic perspective on how shared or divergent cultural models shape the interpretation of metaphorical language. Ultimately, the study provides a foundation for future research on metaphor in globalized, multilingual, and multicultural contexts.

## 2 Literature review

### 2.1 The relationship between culture and metaphor

The interrelation between culture and metaphor has attracted increasing scholarly attention across disciplines such as linguistics, anthropology, and intercultural studies. Metaphor is not only a linguistic phenomenon but also a cognitive and cultural one, deeply rooted in how individuals perceive and structure their world (Kövecses, 2011). As Maalej (2004) argues, metaphor plays a critical role in shaping and reflecting cultural values, offering a powerful tool for decoding cultural worldviews.

Building on the foundational work of Lakoff and Johnson (1980), researchers such as Sharifian (2017) have highlighted the universal grounding of basic metaphors in bodily experience, while also acknowledging the significant variation introduced by cultural schemas. This duality positions metaphor as both a bridge for mutual understanding and a potential site of misunderstanding in intercultural communication.

In China, scholars have conducted comparative analyses of metaphor in English and Chinese, revealing key differences in metaphorical structures and values between Eastern and Western cultural traditions (Pan, 2012; Gao, 2016). These studies underscore the interplay between language and cultural cognition, showing how metaphor reflects and reproduces culture-specific values.

Furthermore, Liu and Shen (2009) demonstrate how metaphors used in Chinese media reflect and respond to socio-cultural shifts, positioning metaphor as a dynamic indicator of cultural change. Similarly, Sun and Wang (2021) and Zhu (2015) argue that metaphor is embedded within cultural cognition and plays a vital role in transmitting cultural knowledge, thereby enriching communication and deepening cross-cultural understanding.

### 2.2 The relationship between cultural patterns and metaphor

Recent scholarship has also emphasized the role of cultural patterns—shared values, beliefs, and practices—in shaping metaphorical cognition. Deignan (2003) suggests that metaphors not only reflect but also help construct cultural patterns. Yu (2008a, 2008b, 2009, 2017) further develops this idea by proposing that metaphor arises from the interaction between bodily experience and culturally shaped cognitive frameworks, emphasizing the inseparability of language, body, and culture.

Chinese scholars have contributed to this discourse by linking traditional cultural concepts with metaphorical expression. For instance, Zhang (2013) explores how metaphors encapsulate cultural patterns such as collectivism, harmony, and relational thinking. Zhao (2015) discusses how classical Chinese ideas like Zhong Yong (中庸, the Doctrine of the Mean) influence metaphorical thinking. Sun (2020) highlights how folk stories serve as repositories of metaphorical expression, laden with cultural meanings.

Nevertheless, existing research often lacks clarity on the mechanisms through which cultural patterns influence metaphor generation and interpretation. While Yu’s body of work has provided a foundation for understanding this interaction, more work is needed to elucidate how different types of cultural models (e.g., high-context vs. low-context cultures) shape metaphorical cognition. Moreover, inconsistencies in the definition of “culture” across studies present methodological challenges, hindering theoretical integration and cross-study comparison.

### 2.3 Metaphor and intercultural communication

Despite significant progress in metaphor and culture research, relatively few studies have focused explicitly on metaphor as a challenge and resource in intercultural communication. Musolff (2014) argue that metaphor can both facilitate and obstruct communication across cultures, depending on whether interlocutors share the cultural schemas underpinning metaphorical expressions (Wu, 2018). Cultural differences may lead to divergent metaphor interpretations, resulting in misunderstanding, stereotyping, or even cultural offense.

In cross-cultural encounters, metaphors often function as implicit cultural codes. When interlocutors lack shared metaphorical mappings, the intended meaning may be distorted or lost. For instance, metaphors of “family” in corporate culture may convey warmth and unity in one context but hierarchy and control in another. This highlights the need for a more systematic investigation into how cultural models affect metaphorical meaning-making across linguistic and cultural boundaries.

### 2.4 Research gaps and questions

To date, most studies have either focused on metaphor’s cultural specificity or its cognitive universality, but few have attempted to integrate these perspectives into a coherent model that explains how cultural models shape metaphor understanding across cultures. Additionally, there is a lack of grounded empirical research that systematically identifies key cultural elements influencing metaphor comprehension.

This paper addresses these gaps by asking:

What are the key cultural elements that influence metaphor understanding across different cultures?

How do these elements interact to shape cross-cultural metaphor comprehension?

To what extent can a grounded theory approach help uncover the dynamic relationship between cultural models and metaphor interpretation?

By investigating these questions, this study seeks to contribute to both the theoretical development and practical application of metaphor research in intercultural contexts.

## 3 Research design

This section elaborates on all components of the research design, including the methodological framework, data sources and collection, coding and data analysis procedures, and theoretical foundations. It adopts a grounded theory approach to explore the mechanisms through which cultural models influence metaphorical understanding in cross-cultural contexts. The design is structured to address the following research questions:

What are the key cultural elements that influence metaphor understanding across different cultures?

How do these cultural elements interact to shape metaphorical comprehension in cross-cultural communication?

How can grounded theory be used to construct a theoretical model explaining the influence of cultural patterns on metaphor interpretation?

### 3.1 Research methodology

Grounded Theory, a methodology rooted in data-driven research, offers flexibility in adjusting research directions. Its primary goal is to generate new theories through in-depth qualitative analysis, particularly in social sciences, when there is a lack of clear theories, existing theories are insufficient to explain certain phenomena (Charmaz, 2006). Originally proposed by Glaser and Strauss in 1967, Grounded Theory is characterized by its qualitative nature and is particularly useful when dealing with complex social phenomena. In this study, Grounded Theory is selected for three main reasons:

Lack of a comprehensive theoretical framework: While there is substantial research on metaphor and culture, few models systematically explain how cultural models shape metaphorical understanding. Grounded theory provides a rigorous pathway to construct such a framework (Charmaz, 2006).

Flexibility and responsiveness to complex data: Cultural metaphor usage varies subtly across linguistic, cultural, and social contexts. Grounded theory allows researchers to flexibly adjust research directions as new patterns emerge (Hall and Callaghan, 2005), making it ideal for capturing these nuances.

Suitability for socio-cultural exploration: Metaphors reflect more than language—they embody cultural cognition, historical values, and societal ideologies. Grounded theory enables the in-depth excavation of these layers and supports the development of an integrated theoretical model.

**Figure 1:**
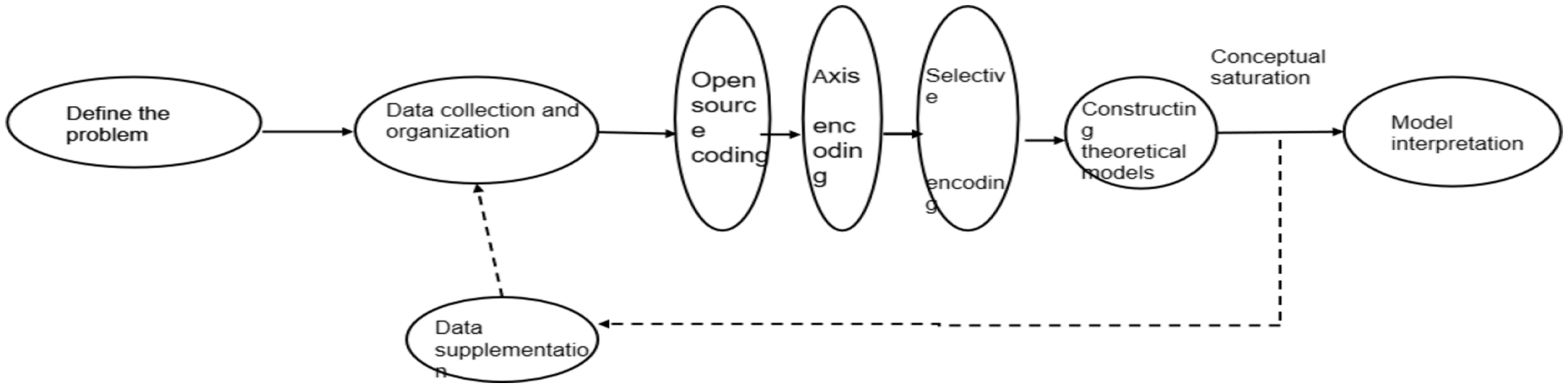
Research process flowchart.

This study aims to extract practical experiences from domestic and international research on the relationship between cultural patterns and metaphor from textual materials. The goal is to elucidate the key elements and theoretical logic through which cultural patterns influence metaphorical understanding. The research steps are illustrated in Figure 1.

### 3.2 Data sources and collection

To address the research questions, data will be collected from the Corpus of Contemporary American English (COCA), the Chinese Corpus Linguistics (CCL), and the China National Knowledge Infrastructure (CNKI). These corpora have been selected for their large-scale nature, diverse sources, and coverage of a wide range of topics (Davies, 2008). Utilizing these corpora will provide comprehensive and accurate language data, facilitating in-depth analysis of language use in English and Chinese. The collected data will include literary works, folk stories, advertising media discourse, religious philosophical texts, legal and political texts, social media network texts, and academic papers, as well as cross-cultural comparative materials related to studies on the relationship between cultural models and metaphor. Literary works, folk stories, and similar materials can reflect the uniqueness and core values of a culture (Hua, 2019), while advertising media discourse and social media texts can depict the modern aspects and esthetic standards of a culture (Li and Guo, 2023). Legal and political texts and academic papers provide tools and perspectives for in-depth cultural analysis (Li, 2018), and cross-cultural comparative materials reveal commonalities and differences between different cultures (Yan and Zhao, 2023). These materials collectively offer rich data support for analyzing how cultural patterns influence metaphorical understanding.

During the literature selection phase, keywords such as “culture,” “cultural patterns,” “metaphor,” and “cross-cultural communication” will be used for retrieval. The literature will be screened based on titles, abstracts, and full texts, applying criteria such as relevance to the research questions, focus on the relationship between cultural patterns and metaphor, and the inclusion of valuable insights into cross-cultural communication. A total of 146 relevant texts from both domestic and international sources were screened. From these, 108 texts were randomly selected for coding analysis and model construction, forming a material repository for open coding. The remaining 38 texts will be reserved for testing theoretical saturation. Through in-depth reading of the full texts, key elements influencing metaphorical understanding due to cultural patterns were identified, and these elements were summarized, categorized, and coded, as presented in Table 1.

**Table 1:** Literature classification table.

Literature focus on cultural patterns and metaphor relationship	Quantity
Literary works	40
Advertising media discourse	16
Religious philosophical texts	10
Legal and political texts	15
Social media network texts	20
Academic papers	30
Cross-cultural comparative materials	15

## 4 Coding analysis and model construction

### 4.1 Open coding

Open coding (Glaser and Strauss, 1967) is the first phase of categorizing, summarizing, and conceptualizing textual data. During this stage, the coding process involves three steps: (1) Import all textual data into Nvivo 12 software and read through them word by word and sentence by sentence. (2) Abstract sentences and cases during the reading process, followed by numbering and naming. (3) Induct similar cases, initiating conceptual coding for them. A total of 108 original statements were collected in this study and abstracted during the coding process. Through coding and classification of these original statements, 18 initial concepts were distilled, and similar initial concepts were synthesized. For example, from the original statements about the constructive role of metaphor in understanding cultural patterns and the foundational role of cultural models in bodily experiences, the initial concepts of “constructive role” and “foundational role” were extracted. They were categorized under one label: Cultural Influence. The extraction of initial categories is a crucial step in the Grounded Theory research process, aiding a better understanding of research questions, discovering latent themes and concepts, forming a research framework, and enhancing the reliability and effectiveness of the theory. Consequently, a total of 9 initial categories were identified, as shown in Table 2.

**Table 2:** Examples of open coding.

Initial categories	Initial conceptualization	Excerpts from original data
A1 Cultural influence	a1 Constructive Role	Metaphors shape abstract cultural concepts; in the U.S., success is metaphorically climbing a pyramid toward the peak, each step signifies progress, while in Japan, it’s like a continuous, gentle effort and resilience.
	a2 Foundational Role	Cultural patterns play a central role in conceptualizing bodily experiences; in France, love is metaphorically like aged wine, becoming more aromatic over time, while in India, it’s akin to a sacred tree in the garden, requiring rituals and devotion.
A2 Cultural pattern differences	a3 Cognitive Metaphor Differences	Universal bodily experiences may result in different conceptual metaphors interpreted by different cultural patterns; in Spain, life is metaphorically a passionate dance, full of vigor and vitality, while in China, it’s like an elegant ink painting, emphasizing harmony and balance.
	a4 Core Metaphor Differences	Differences in Eastern and Western cultural patterns and how they determine dominant metaphors in a language; in the U.S., time is often seen as a straight line, symbolizing continuous progress, while in India, it’s more like a cycle, signifying rebirth and the endless cycle of the universe.
A3 Bodily experience	a5 Embodied Metaphors	Warmth is often associated with love, passion, and comfort, while cold is linked to distance, indifference, and disappointment; for example, in Chinese, there is a distinction between “warm-hearted” and “cold-hearted,” similar distinctions exist in English like “warm moments” and “cold-hearted.”
	a6 Gustatory Metaphors	Describing pleasant love in Chinese as “sweet” and hardships as “bitter”; similarly, in English, expressions like “sweet moments” and “bitter experience” convey these metaphors.
A4 Cultural consensus	a7 Cognitive Construction	Different cultural patterns conceptualize mental activities using different body parts; in Arabic culture, fate is metaphorically seen as a woven carpet, each thread predestined by the divine, while in the U.S., fate is more like an unpaved road, shaped by individual choices.
	a8 Ethnic Culture	Metaphors embodying deep ethnic cultural traditions and values; in Navajo culture, land is seen as a nurturing mother, the source of life and protection, while in the U.K., land is more metaphorically the cornerstone of the kingdom, representing power and rule.
A5 Metaphorical function	a9 Abstract Concepts	In the West, knowledge is often viewed as a weapon for competition and conquest; in the East, it’s more like a seed, requiring patient nurturing to yield wisdom.
	a10 Metaphorical Vocabulary	In Japan, harmony is likened to a lotus flower in the garden, blooming independently even in murky waters; in France, harmony is more like meticulous clockwork craftsmanship, ensuring each part fits precisely for the smooth functioning of the whole.
A6 Cultural metaphors	a11 Metaphor Uniqueness	Specific cultural patterns determine the referent used in metaphor, leading to unique metaphors; in Maasai culture, courage is metaphorically the heart of a lion, symbolizing fearlessness and strength, while in Norway, courage is more like a solid ice layer, unaffected by the harsh cold, quietly enduring.
	a12 Symbolism of Culture	Symbolic language expressions in metaphors associating one concept with another, creating deeper cultural meanings; e.g., “Our team is like a ship navigating stormy seas,” where the ship represents the team and the ocean symbolizes challenges, change, and uncertainty.
A7 Metaphorical differences	a13 Metaphor Variability	Comparing how English and Chinese express mental and emotional activities using “head” and “heart”; in Eastern culture, time is often described as a slow-flowing river, signifying its slow yet continuous passage, while in Western culture, time is more like a rapidly pulsating rhythm, each moment appearing urgent and forceful.
	a14 Metaphor Diversity	English metaphors and Chinese “譬喻” not only share commonality but also exhibit individuality; in China, love is often metaphorically described as a continuous stream, representing sustained commitment and care, while in Brazil, love is often seen as a passionate samba dance, full of intensity and vitality.
A8 Cultural interaction	a15 Conceptual Cognition	Understanding abstract concepts is rooted in the interaction of human experience with the real world; in India, life is metaphorically a journey of continuous cycles, emphasizing destiny and recurrence, while in Canada, life might be perceived more as a path to explore the unknown.
	a16 Shared Cognitive	Similar cognitive patterns regarding appropriate etiquette and politeness across cultures; for instance, handshakes, bows, greetings are universally seen as expressions of respect and politeness. This shared cognitive pattern enables individuals from different cultures to understand each other’s social expectations.
A9 Cultural experience	a17 Cultural Factors	Examining cultural factors in the interpretation of metaphors, such as wealth; in Eastern culture, prosperity is metaphorically a lush tree providing shade to its surroundings, while in Western culture, prosperity might be more like a towering skyscraper, showcasing power and success.
	a18 Traditional Rituals	Participation in traditional cultural rituals, festivals, religious ceremonies, etc., allowing individuals to personally experience the charm and importance of cultural traditions; for instance, participating in a traditional cultural festival enables individuals to not only feel the atmosphere of the celebration but also understand cultural values, attitudes toward family, and the significance of food.

### 4.2 Axial coding

The primary task of axial coding (Glaser and Strauss, 1967) is to summarize and consolidate initial categories based on open coding. It involves identifying commonalities between concepts, establishing connections among various concepts, conducting in-depth analyses of each concept and category based on the interrelatedness and logical coherence of different categories within a concept. Concepts that cannot continue to converge into categories with other concepts are eliminated, and the remaining categories are adjusted. It is crucial to repeatedly read and scrutinize data relevant to each research category to check for the emergence of new concepts or theoretical constructs.

This process ensures the exclusivity and rigor of each category, meaning that each category should have a clear definition and boundaries to avoid overlapping and fuzziness of concepts. This safeguards the logical consistency and reliability of research conclusions, thereby forming main categories. For instance, by interpreting the analogous connotations of “Cultural Pattern Differences” and “Cultural Metaphors” within the initial categories, we derived the main category “Cognitive Patterns” based on the understanding that both “Cultural Pattern Differences” and “Cultural Metaphors” belong to human cognitive patterns. Through axial coding, we further distilled four main categories from the initial nine categories, as illustrated in Table 3.

**Table 3:** Main categories formed through axial coding.

Main categories	Initial categories	Conceptual explanations
A1 Cognitive patterns	a1 Cultural Pattern Differences	Differences in thinking patterns, values, and behavioral norms among different cultures. These cultural pattern differences influence people’s thinking and behavior habits.
	a2 Cultural Metaphors	Using culture-specific metaphors to express concepts, making analogies between one concept or thing and another for better understanding or interpretation. Cultural metaphors form within specific cultures and are closely related to cultural values, beliefs, and customs.
A2 Cognitive frameworks	a3 Cultural Impact	Culture plays a crucial role in shaping individual and societal cognition, behavior, and organization. It provides a set of shared values and behavioral norms, influencing decision-making, behavior, and social interactions.
	a4 Metaphorical Features	Metaphors, as a rhetorical device, transform abstract concepts into concrete images through comparison and symbolism. This enhances the emotional impact and expressiveness of works, facilitating the conveyance of the author’s intent and viewpoint.
A3 Language communication	a5 Cultural Interaction	The process of communication, interaction, and mutual influence between different cultures. People engage in direct communication and interaction with individuals from different cultures through activities such as travel, work, and studying abroad.
	a6 Metaphorical Differences	Differences in the expression and understanding of metaphors across languages and cultures. Respecting and understanding the ways they interpret and use metaphors are crucial in cross-cultural communication.
A4 Social consensus	a7 Embodied Experience	Cognition and understanding of external objects and one’s own state through sensory and bodily experiences. It is a way in which we interact with and perceive the world, forming a social consensus.
	a8 Cultural Consensus	Shared understanding and accepted viewpoints within specific cultural groups regarding cultural elements, values, behavioral norms, traditional customs, etc. These consensuses result from cultural inheritance and social learning, reflecting the cultural group’s sense of identity and cohesion.
	a9 Cultural Experience	An essential way individuals interact and communicate with culture. It enables individuals to better understand and respect different cultures, fostering the inheritance and development of cultural diversity.

### 4.3 Selective coding

**Figure 2:**
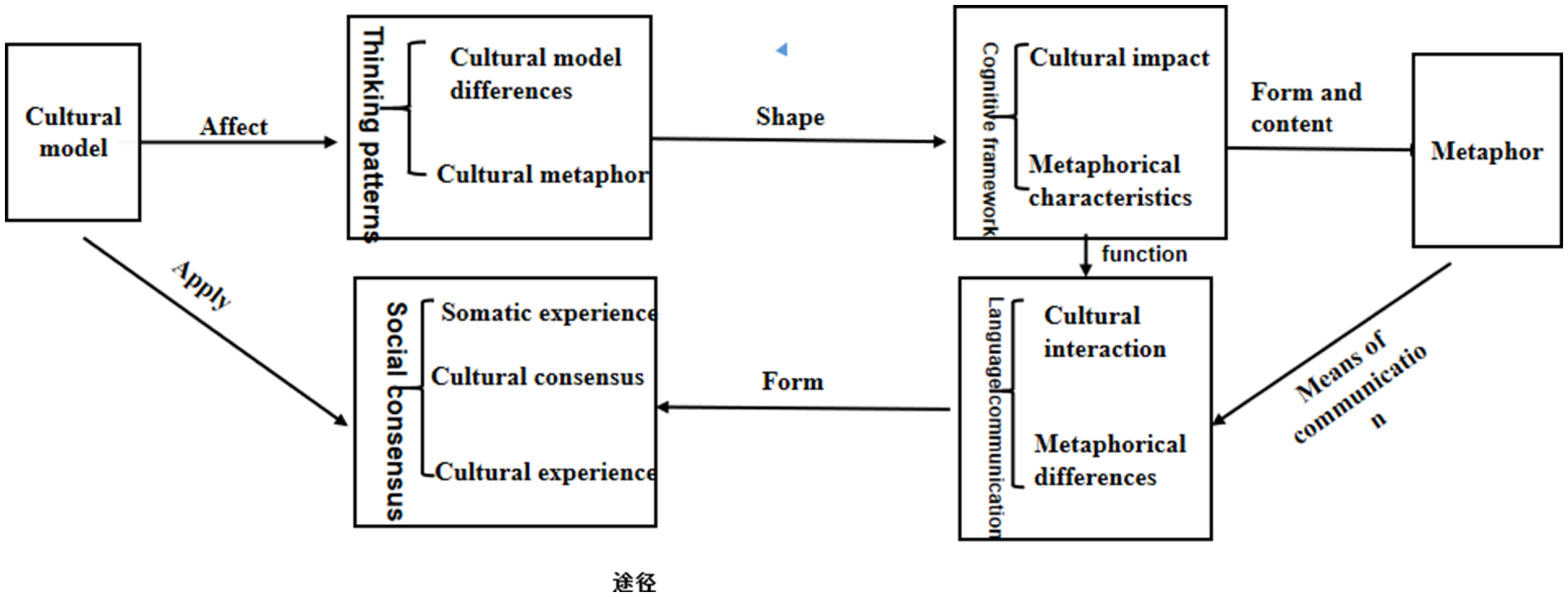
Theoretical framework based on core category and main categories.

Glaser and Strauss (1967) identified selective coding as a core process focusing on connecting various categories identified in open coding to their core category to form an integrated theory. Selective coding involves explicitly distinguishing between primary and secondary categories, describing the data around the “core category,” which refers to the most important and frequently occurring concept or category emerging from the data in grounded theory research. The core category serves as the central focus for the study, connecting all other minor categories in the research. It provides a central focus and guides subsequent data collection and analysis (Glaser and Strauss, 1967). Leveraging well-developed main categories, this paper elucidates the entire “storyline” to form a theoretically relevant framework, as illustrated in Figure 2.

According to the research paradigm of grounded theory and systematic analysis, this paper identifies the key elements influencing metaphorical understanding as thinking patterns, cognitive frameworks, language communication, and social consensus. The “storyline” can be summarized as follows: cultural patterns influence individual thinking patterns, and metaphors, based on cultural patterns, further shape and express thinking. People’s thinking patterns influence the formation of cognitive frameworks, and different cognitive frameworks, in turn, affect language communication. The resulting social consensus, in interaction, influences both cultural patterns and metaphors.

### 4.4 Saturation test of the theoretical model

To ensure the scientific rigor of the grounded theory research process and the accuracy of the results, this paper first conceptualizes the implicit correlations between concepts or categories formed through open coding and axial coding (Glaser and Strauss, 1967). Secondly, by using the same research methods such as coding and analysis, the remaining 38 texts are subjected to a saturation test of the theoretical model. Saturation (Glaser and Strauss, 1967) refers to reaching a point in the data collection and analysis process where additional data no longer provide new insights or add new codes and concepts, indicating that saturation has been achieved.

After comprehensive coding analysis of the selected literature, no new main categories were obtained, except for thinking patterns, cognitive frameworks, language communication, and social consensus. All newly discovered aspects were encompassed by the 4 main categories previously extracted. Therefore, this paper considers the preliminary establishment of selective coding to be theoretically saturated.

## 5 Results and discussion

This section presents and discusses the findings based on the analytical model developed in this study. It is structured into five key thematic categories: thinking patterns, cognitive frameworks, language communication, social consensus, and their mechanisms of influence on metaphorical understanding. Each subsection addresses specific research questions and compares the findings with relevant literature to situate this study within broader scholarly conversations.

### 5.1 Thinking patterns

Research question addressed: How do cultural thinking patterns influence the understanding and use of metaphors?

Thinking patterns are core cognitive frameworks that individuals adopt when interpreting the world. Our findings indicate that these patterns significantly influence metaphor identification and application, aligning with Smith (2001) and Taylor (1995), who argue that thinking patterns mediate cultural meaning through metaphor (Bruner, 1990; Fauconnier and Turner, 2002).

For example, the Western metaphor “time is money” reflects a linear, individualistic mode of thought rooted in efficiency and productivity (Hofstede, 1984). In contrast, Eastern interpretations of time often align with relational and cyclical thinking, emphasizing continuity and interpersonal harmony. This supports and extends the work of Hall (1976) on high-context cultures, by showing how these patterns manifest metaphorically.

However, our analysis also revealed a lack of empirical data supporting these associations. While the patterns observed are consistent with established cultural frameworks, future work should incorporate cognitive interviews or metaphor elicitation tasks to provide more rigorous evidence.

### 5.2 Cognitive frameworks

Research question addressed: What is the role of cognitive frameworks in shaping metaphor interpretation across cultures?

Cognitive frameworks—comprising beliefs, experiences, and values—serve as the mental scaffolds through which individuals interpret metaphors (Lakoff and Johnson, 1999). The data align with Johnson (1987) and Lakoff and Johnson (1980), suggesting that these frameworks are not only shaped by culture but also shape metaphorical comprehension.

For instance, Western emphasis on efficiency results in metaphors such as “time is money,” whereas Eastern values lead to metaphors like “time is a flowing river,” suggesting continuity and patience. These findings resonate with existing theories but highlight an underexplored area: Littlemore (2019) also notes individual variation in metaphor use due to differences in embodied experiences and cultural exposure. How multiple cultural influences (e.g., globalization or bilingualism) might hybridize or shift such frameworks. More longitudinal or cross-generational studies would help investigate this dynamic.

### 5.3 Language communication

Research question addressed: In what ways does language communication mediate metaphorical meaning across cultural boundaries?

Language is a primary vehicle for transmitting cognitive and cultural patterns. Consistent with Whorf (1956), the study found that metaphors reflect not only linguistic structure but also embedded cultural values. Examples such as “love is a flowing stream” in Chinese and “love is a samba” in Brazilian Portuguese illustrate how metaphor shapes emotional expression differently across cultures.

This supports the theory of linguistic relativity and extends earlier works on intercultural communication, including Gudykunst (2003) and Gudykunst and Kim’s (2003), by showing how metaphorical mismatches can cause misunderstanding in cross-cultural settings (Hall, 1959). Chen (2002) highlights similar East-West contrasts in intercultural dynamics. Nevertheless, the current analysis lacks data from authentic interaction contexts (e.g., recorded conversations), which would provide a stronger basis for examining how metaphors are used in real-time communication.

### 5.4 Social consensus

Research question addressed: How does social consensus influence the interpretation and acceptance of metaphors in different cultures?

The findings show that social consensus—shared values and collective agreements—shapes metaphor comprehension by anchoring it in bodily experience and cultural norms. For instance, metaphors like “harmony is precious” (Eastern) and “survival of the fittest” (Western) demonstrate how different social values produce divergent metaphorical imagery, consistent with Yu (2008a, 2008b, 2009, 2017) and Jia (2016). See also Yu (2015), who emphasizes the embodied nature of culture in shaping metaphorical systems.

This underscores how metaphors are not merely linguistic expressions but social acts that reinforce or challenge cultural norms. However, more empirical data on how individuals adopt or resist dominant metaphors within their culture is needed to deepen this analysis.

### 5.5 Mechanisms of their influence on metaphorical understanding

Research question addressed: How do thinking patterns, cognitive frameworks, language, and social consensus collectively influence metaphorical understanding?

This integrative section shows that metaphorical understanding emerges from the dynamic interplay of thinking patterns, cognitive frameworks, language communication, and social consensus. Thinking patterns shape cognitive frameworks, which are articulated and negotiated through language and reinforced by social consensus.

This supports an ecological model of metaphor understanding, where individual cognition and social structures co-construct meaning. Mischler (2013) supports this view by exploring the temporal and cultural interplay of embodied metaphor. While conceptually robust, the model currently lacks empirical grounding. Future studies should explore how these dimensions interact using mixed methods, such as combining discourse analysis with surveys or experimental tasks.

## 6 Conclusion

### 6.1 Research findings

Based on grounded theory, a theoretical model of the key elements influencing metaphorical understanding, was constructed: Cultural Models Influence Metaphorical Understanding. Thinking patterns establish cognitive frameworks, which are clarified and shared through language communication, thereby establishing common understanding within social groups, known as social consensus. They play significant roles in the understanding of metaphors in cross-cultural communication.

Thinking patterns shape our cognitive frameworks, which are manifested in language interactions. Social consensus, influenced by cultural patterns, is further solidified through language communication. Thinking patterns and cognitive frameworks lay the foundation for metaphor formation, and language communication provides channels for dissemination. Social consensus creates conditions for the acceptance and sustained existence of metaphors. These elements interact, collectively shaping the cognitive and applied ways individuals and cultural collectives interpret metaphors (Boroditsky, 2011).

### 6.2 Research contribution

This study contributes to theoretical research by integrating and analyzing existing literature, extracting key elements of “Cultural Models Influence Metaphorical Understanding,” and constructing a theoretical model. The aim is to provide a theoretical framework for efficient across cultural communication, this reduces misunderstandings and errors, and offering reference and guidance for future research and practice.

### 6.3 Limitations and prospects

The data for this study come from a variety of sources including, folk stories, media content, religious and philosophical texts, legal and political documents, social media texts, academic papers, and cross-cultural comparison materials related to “Cultural Models Influence Metaphorical Understanding.” Although efforts were made to consider the comprehensiveness and completeness of the data during the collection and coding process, following the principles of theoretical saturation, the literature used may still have a certain degree of bias.

To enhance the validity and reliability of the theoretical model proposed, future research could incorporate in-depth interviews with participants from various cultural backgrounds. Such qualitative data would provide rich, contextual insights into how individuals use and interpret metaphors in everyday communication. Additionally, the Delphi method may be employed in future studies to systematically gather expert consensus on the categorization and interpretation of metaphor-related data, thus improving the objectivity of analysis and reducing researcher bias.

This study opens new avenues for further investigation into the application of metaphorical understanding in fields such as education, translation, and artificial intelligence. For instance:

In education, understanding how metaphors vary across cultures could inform culturally responsive teaching practices, curriculum design, and language instruction, particularly in multilingual classrooms.

In translation studies, insights into cultural metaphors could enhance translation accuracy and intercultural readability, especially for literary, religious, and legal texts that are rich in metaphorical language.

In the realm of artificial intelligence, metaphor-aware natural language processing (NLP) models could be designed to better interpret and generate culturally sensitive language, improving cross-cultural communication in machine-human interaction.

Future studies may explore how metaphorical structures are processed differently in multilingual individuals, or how global digital culture might be shaping new, hybrid metaphors that transcend traditional cultural boundaries. Interdisciplinary collaboration with fields such as cognitive neuroscience, AI ethics, and intercultural pedagogy may further deepen our understanding of metaphor in a rapidly changing world.

## Data Availability

The original contributions presented in the study are included in the article/supplementary material, further inquiries can be directed to the corresponding author.
